# Reduction of pesticide application via real-time precision spraying

**DOI:** 10.1038/s41598-022-09607-w

**Published:** 2022-04-04

**Authors:** Alex Rogers Aguiar Zanin, Danilo Carvalho Neves, Larissa Pereira Ribeiro Teodoro, Carlos Antonio da Silva Júnior, Simone Pereira da Silva, Paulo Eduardo Teodoro, Fábio Henrique Rojo Baio

**Affiliations:** 1 Universidade Federal de Mato Grosso do Sul (UFMS), Chapadão do Sul, MS Brazil; 2 Universidade do Estado de Mato Grosso (UNEMAT), Sinop, MT Brazil

**Keywords:** Plant sciences, Environmental sciences, Environmental social sciences

## Abstract

Farmers focus on reducing the cost of production and aim to increase profit. The objective of this study was to quantify the reduction of pesticides applied to soybean (*Glycine max* (L.) Merrill) and maize (*Zea mays* L.) crops in several stages of the production cycle using a site-specific spraying application based on real-time sensors in the Brazilian Cerrado region. The sprayers were equipped with a precision spraying control system based on a real-time sensor. The spraying operations were performed not only for herbicide, but also for fungicide and insecticides applications. The maps recorded the percentage of the spray boom when the application was turned on (on/off spray system) with nozzle-to-nozzle control. The precision spraying system based on real-time sensors reduced the volume of pesticides (including herbicides, insecticides, and fungicides) applied to soybean and maize crops. There was a more significant reduction in the volume of pesticides applied post-emergence of the crops in the initial stages of soybean and maize when the crops had less leaf area or less foliage coverage between the rows. The cost reduction achieved using this technology was 2.3 times lower than the cost associated with pesticide application over the entire area using a conventional sprayer. Under the experimental conditions, there were no differences in the average crop yield, compared to the historical productivity of soybean and maize crops by applying this technology because the recommended doses were not affected and the site of application was limited to points where the presence of plants was present was detected.

## Introduction

The majority of crop prices vary depending on supply and demand on the market. The only way for farmers to increase profitability is to decrease production costs without compromising crop yield or increasing the risk of the activity. Precision agriculture is aimed at increasing the efficiency of using machines and inputs to improve yield and profitability and decrease environmental impact^1^. Precision agriculture allows an improvement in agricultural production management, using practices within the field, and requires an accurate assessment of spatial variations in short scales of resolution^2^.

Humanity requires ecological management practices with a decrease in the volume of pesticides used in farm fields. Accordingly, there are three possible ways to reduce the volume of pesticides used^3^: reducing the number of spray applications during the cropping period, which can compromise crop yield; decreasing the applied dose, which can decrease the level of control and promote the appearance of genetic resistance; and restricting the treated area. The third strategy is the basic principle of site-specific application of pesticides using precision agriculture techniques^4^.

This technology has been applied in several agricultural inputs (e.g., while using herbicides), as there is spatial variability in the growth of weeds in the field^5^. The management of weeds according to the spatial variability of their growth reduces the use of herbicides due to application in areas where the infestation is above the level of the economic threshold^6^. The misuse of herbicides is related to at least three main problems: the application of herbicides at an inadequate phenological stage, herbicide application without considering the infestation level, and application of herbicides in places free of weeds^7^. The first and second problems can be solved via application of technical knowledge by the farmer or consultant; however, strategic planning and tools are feasible for solving the third problem. Thus, one possible alternative would be weed management using precision spraying techniques, thereby potentially reducing the volume of herbicides applied to the soil and the environment^8^.

Currently, the site-specific application of pesticides focuses mainly on herbicides^9^; however, the same technique can be used for other pesticides, such as fungicides^3,10^. The site-specific fungicide application measures the dose across the field surface according to the canopy density regarding preventive control strategies. Denser canopies receive a higher dose of fungicide via this strategy, assuming that a dense canopy creates a microclimate that is more conducive to diseases and contributes significantly to crop yield^3^. Studies about site-specific insecticide application are less prevalent than those about herbicides because the population dynamics of flying insects are very intense^11^. However, this type of site-specific management is also possible for insects, as long as the treated mapped area is bordered as a safe area for proper control^12^.

Application of the theory of site-specific application of pesticides depends on mapping the spatial variability of the targeted crop problem (weed, disease, crop foliar mass, or insects). Site-specific weed control is more common and can be performed using two primary methodologies: weed mapping, followed by preparation of a prescription map containing volume rates of herbicide; and mapping and application in real-time^9^.

Weeds have an irregular distribution (irregular growth in terms of phenology and density), implying that some field areas are below the economic threshold level. However, the enormous challenge is to map the weed spatial variability in large areas in a short time (days) because they commonly proliferate rapidly in the field and can render the generated maps unusable^4^. The use of remote sensing tools can assist farmers in obtaining maps in a short time. There are several ways to map the spatial variability of weeds using remote sensing, which can be done either by satellite images, by unmanned or piloted aircraft, or by real-time detection sensors^13^. However, onboard satellite sensor platforms are often unsuitable for mapping weeds because of their low spatial and temporal resolution^2^. Studies with unmanned aircraft carrying cameras have been developed for weed mapping^14^; however, some processing time of the images in the laboratory makes the process impractical for its application in large areas.

Weed mapping using cameras still faces the problem of differentiation between weeds and crops because of the similarity between their spectral signatures^15^. Differentiating between plants and soil is relatively easy, as their spectral signatures are distinct; however, differentiating different plant species is not a simple process and often requires more expensive sensors, such as hyperspectral sensors^16^. Another strategy for performing the site-specific application of pesticides is through the use of sensors in real-time. The use of real-time sensor equipment for the instant detection of plants offers advantages in its use because of the spraying application at the same time as the target mapping, in addition to the advantage of performance in a single operation, without labor costs, data analysis, and the aid of a prescription map^15^. A study by Biller et al.^17^ was among the first studies with a focus on site-specific spraying of herbicides in real-time and proposed using electronic sensors to detect weeds. The sensors can distinguish the green color of the plants and the soil once plants have different reflectance characteristics. Dammer and Wartenberg^5^ achieved a reduction of 12.7% to 40.9% using site-specific weed control, compared to conventional spraying in the total area.

Methodologies for site-specific treatments have been developed to determine the spatial variability of plants and are mainly associated with weeds. However, using this equipment with sensors in real-time can detect and map weeds and the variability of planting patterns of commercial crops, i.e. crop rows and interrows. Thus, the focus of the current study was the evaluation of site-specific spraying application of pesticides other than herbicides. The current study aimed to quantify the reduction of pesticides applied to soybean and maize crops in several stages of the production cycle using a site-specific spraying application based on real-time sensors in the Brazilian Cerrado region.

## Materials and methods

### Experimental fields

**Figure 1:**
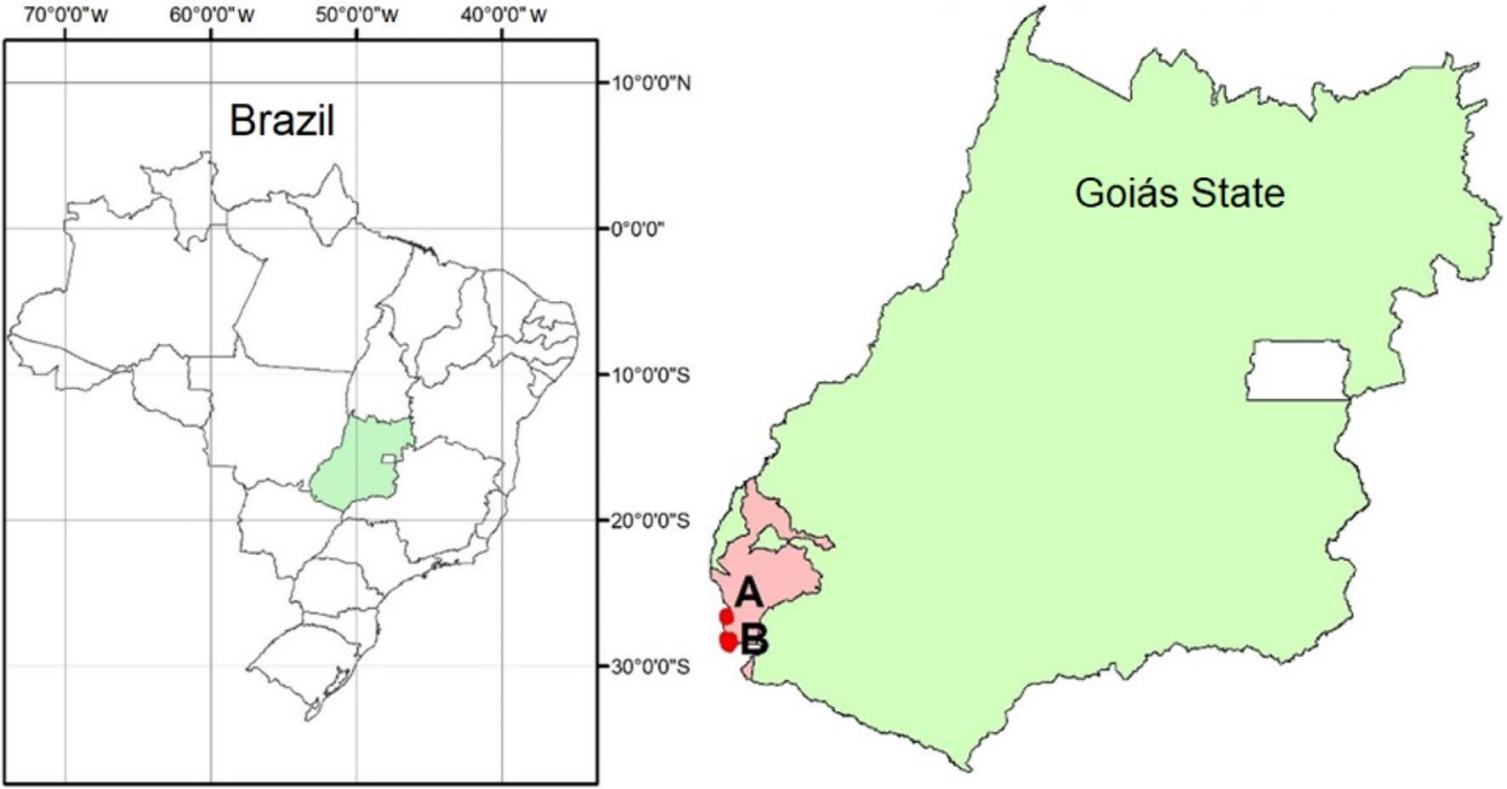
Farms where the experiments were installed comparing conventional and real-time precision spraying, during 2019 and 2020 harvest seasons (GO, Brazil): (**A**) site 1 and (**B**) site 2. The sofware used to create this Figure was ArcGIS Desktop (v10.5, https://desktop.arcgis.com/en/system-requirements/10.5/).

The data analyzed were from pesticide applications (herbicides, insecticides, and fungicides) sprayed on soybean (*Glycine max* (L.) Merrill) and maize (*Zea mays* L.) on two farms (Fig. 1), and two crop seasons in the municipality of Mineiros, GO, Brazil. The farm Nova Geração (site 1 with 2642 ha) is located at 18° 29′ 06.26′′ S and 53° 10′ 23.08′′ W, at an altitude of 815 m, and the farm Cristiane (site 2 with 1060 ha) is located at 18° 08′ 21.06′′ S and 53° 10′ 48.18′′ W, at an altitude of 810 m. Both farms were cropped with the no-tillage system, with an average annual precipitation of 1600 mm. Data were collected from a total of 22 fields from both farms. The area of site 1 is divided into 20 fields (ranging from 21 to 262 ha) and that of site 2 was divided into two fields (920 and 140 ha).

The crop genetic materials employed in the cultivation of soybeans were Desafio and Foco varieties from Brasmax™ (Rio Verde/GO, Brazil). These varieties were cropped in the 2018–2019 and 2019–2020 harvest seasons, respectively, as the primary crop. The sowing of soybean on the experimental fields was performed at a row spacing of 0.45 m and with a population of 300,000 seeds ha^−1^. As the secondary crop, in the succession of soybean at the same areas, the maize crop was cultivated using the genetic materials 2A401 PW and CD3612 PW (obtained from the company Brevante Seeds™, São Paulo, Brazil) and was sown at a row spacing of 0.90 m and with a population of 57,000 seeds ha^−1^. The experiments were settled in accordance with international, national and/or institutional guidelines.

### Site-specific spraying equipment

**Figure 2:**
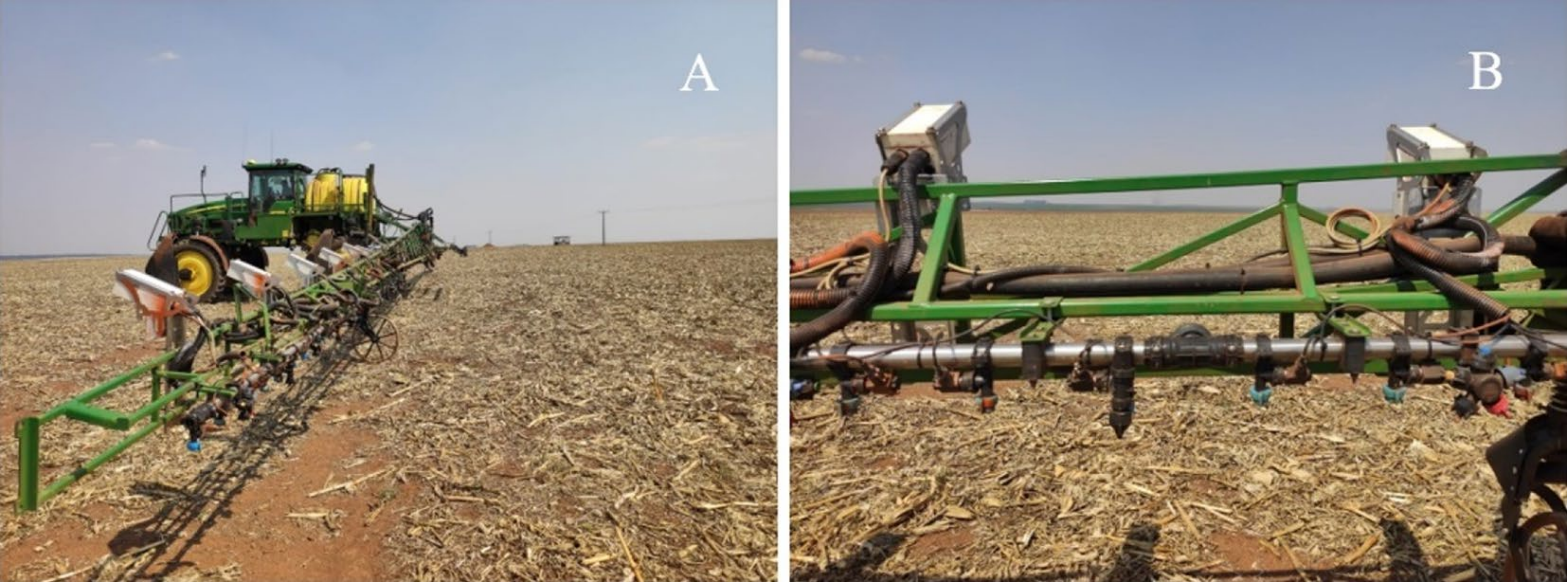
Illustration of the sensors installed on the self-propelled sprayer: (**A**) sprayer boom and sensors; (**B**) sensors positioned in front of the sprayer boom.

Field management using the site-specific spraying application based on real-time sensors was similar between the two farms. Spraying applications were conducted in the entire field of soybean and maize crops. A self-propelled sprayer (John Deere 4730, Catalão, Brazil) was used on each farm. The sprayer was equipped with a spray boom of 36 m and a hydraulic stainless flat fan nozzle (Teejet 4003, Glendale Heights, USA). The nozzle spacing was 0.3 m, and the spray speed was 5.6 m s^−1^. This nozzle can apply a flow rate of 1.1 L min^−1^ at a hydraulic working pressure of 2.8 bar (280 kPa). The site-specific spraying system Weed-it™ (Smart Sensing), with real-time sensors for detecting plants, was assembled on the sprayer (Fig. 2). This equipment contains 36 sensors installed with the sprayer boom, with a spacing of 1 m. Each sensor consists of five independent detection channels, which control five independent spray nozzles. Thus, each sensor covers a width of 0.20 m. These sensors operate at the height of 0.60–0.80 m from the crop canopy.

The real-time sensors facilitate function by detecting the plant, which is followed by instantaneous spraying by opening and closing (on/off method) pulse width modulation (PWM) valves. Each PWM valve controls each spray nozzle on the sprayer boom. The PWM system can operate by changing the application rate; however, the experiment was performed by spraying at a single application rate (100 L ha^−1^). The sensor quantifies the light reflectance from the target (plant, soil, or straw). Plants reflect light with fluorescent properties of chlorophyll molecules present in the leaves of living plants^18^. The active equipment (active sensor, as it has its light source) emits light in the red band, with peak emission at a wavelength of 690 nm. The fluorescence sensor reads at an acquisition rate of 40 Hz from chlorophyll A^19^.

The PWM valves also allow the application rate to be constant, regardless of the displacement speed of the sprayer, owing to the electronic control over the frequency of opening of the valve^20^. This characteristic has the advantage of maintaining a constant hydraulic pressure in the sprayer circuit without changing the size of the sprayed droplet^21^.

Research involving pesticide application technology must characterize the sprayed droplet spectrum, spray nozzle characteristics, and application rate information. The volumetric median diameter (VMD) relative to the droplet size, the relative amplitude (Span index), and the percentage of droplets smaller than 100 μm generated by the nozzle Teejet 4003 were measured using laser diffraction techniques with a Spraytec particle analyzer (Malvern, United Kingdom). The drop size chosen for all applications was medium.

### Spraying applications monitored

The spraying applications were classified into four types of operations for each crop (Table 1): for the soybean crop, weed desiccation, crop pre-planting, first post-emergence application, and crop defoliation (pre-harvesting); for the maize crop, the first, second, third, and fourth applications of post-emergence herbicides. The experiment comparison was performed based on 721 spray applications (Table 1) over 3702 ha of land, over the crop seasons across two years.

**Table 1:** Number of spray applications and type of spray operation on each crop, utilizing real-time precision spraying, during 2019 and 2020 harvest seasons (GO, Brazil).

Operations	Spray appl.	Month	Operations	Spray appl.	Month
**Soybean (principal crop cycle)**			**Maize (second crop cycle)**		
Dissecation	166	September	Sowing		February
Pre-planting	63	October	1st Aplication	141	February
Sowing		November	2nd Aplication	144	February
1^st^ Aplication	27	November	3rd Aplication	97	March
Defoliation	58	January	4th Aplication	25	March
Harvesting		February	Harvesting		April
Total	314		Total	407	

### Analysis of the data collected

The electronic spray controller recorded application maps that were applied for statistical analysis. The maps recorded the percentage of the spray boom when the application was turned on (on/off spray system) with nozzle-to-nozzle control. Thereafter, it was possible to quantify the percentage of the cropped field with each percentage of the spray boom opened.

The statistical design was completely randomized with an unbalanced number of replications. The variables analyzed in each application (total of 721 spraying applications) in each crop and agricultural year were: type of pesticide; quantity of each pesticide applied; unit price of each pesticide; total pesticide applied; treated field area; and total cost per field area.

The volume of the individual pesticide applied to each spray application and field was compared with the volume consumed if the real-time sensor was not present (conventional spraying application) and if the recommended pesticide dose was applied to the entire field. Comparative data were subjected to analysis of variance using the statistical software Rbio^22^. The analyses were divided between soybean and maize crops, and treatments that showed significant differences were subjected to the Duncan test with a probability of 5% for comparison of averages. Box plots were created for the analysis of data variability.

### Comparison of crop yield

The average crop yield of each field from the two agricultural years was compared to the crop yield from 2 years before the installation of the technology, allowing observation of whether the new technique caused any damage to the average yield of each field in the experimental farms (Supplementary Table S1).

### Economic analysis

An economic analysis was performed to calculate the feasibility of the adopted system according to the volume of the agricultural pesticides consumed and the initial investment in the technology. The analysis considered the partial budget methodology, which evaluates the effect of the new technology on the existing production structure^23^. The entire list of pesticides applied was documented during both crop cycles. Each field received a recommendation and dose of pesticides, according to the needs at the time of the crop. Furthermore, all these factors were compared field by field individually for cost and reduction percentage comparative purposes. The following factors were considered in the analysis, described in Table 2. The price of pesticides was quoted in a store in the local market.

**Table 2:** Variables considered in the economic evaluation to compare the conventional and real-time precision spraying, during 2019 and 2020 harvest seasons (GO, Brazil).

Variable	Description
Exchange rate	R$ 5.30 per dollar^24^
Depreciation cost	10% per year at a linear rate
Useful life of the equipment	15 years^23^
Annual interest rate	6.5% per year
Operational field efficiency	75%^25^
Self-propelled sprayer	USD $ 150,000.00
Site-specific spraying equipment	USD $ 220,000.00

## Results and discussion

### Spray application maps

**Figure 3:**
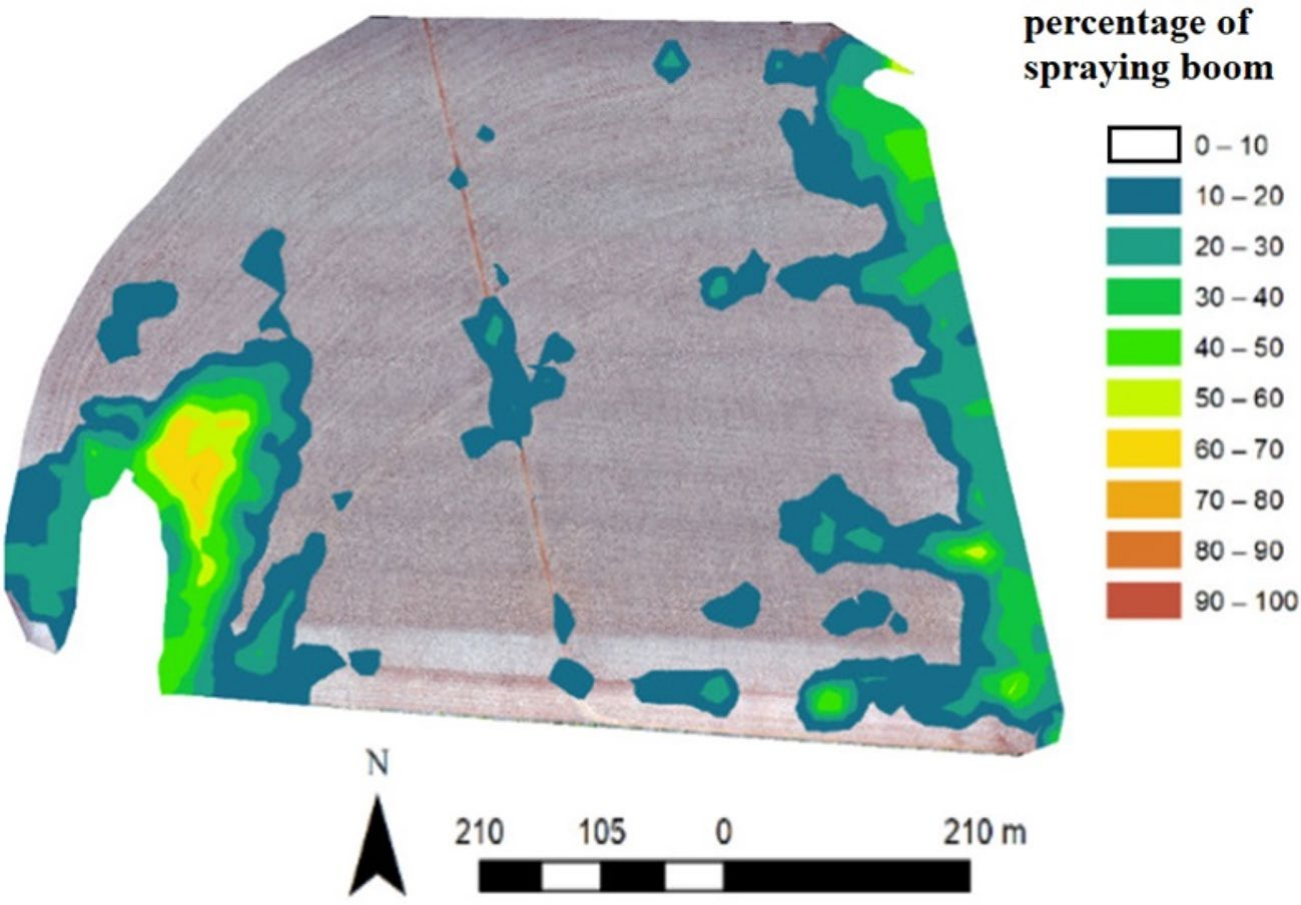
Spatial variability of the sprayer boom application throughout the field in pre-planting operation.

To illustrate that it is possible to apply pesticides only in the necessary locations of the field, Fig. 3 illustrates the spatial variability of the percentage of the sprayer boom that was effectively sprayed throughout the field in a spray of herbicide in the pre-planting operation of soybean crops in one of the evaluated fields. It was observed that weeds occur in patches, with no need for spraying in the entire crop field, which would waste some volume of pesticides and increase the environmental contamination. Some locations in this field required zero or less than 10% of sprayer boom during the operation. If weeds occur in patches, the implementation of site-specific weed management relies on accurate weed monitoring^26^. Sensors on agricultural machines, drones, or high-resolution satellite-based platforms play a central role in this process. Site-specific spraying can reduce herbicide usage by up to 70% while maintaining 100% weed control^27^.

### Spectrum of the droplets sprayed

**Figure 4:**
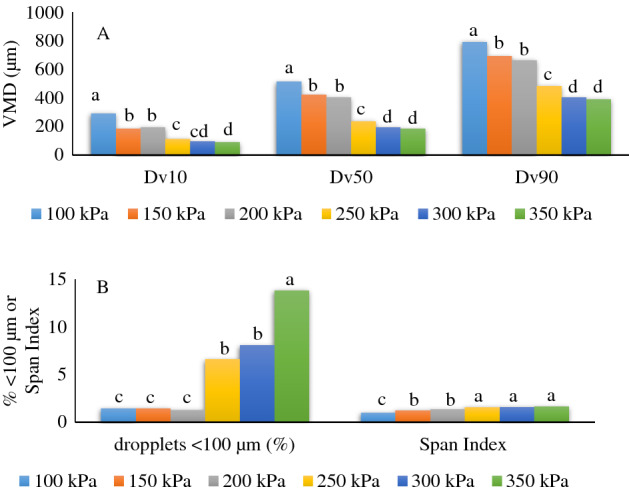
Volume median volumetric of the droplets formed by the flat fan nozzle Teejet 4003 (**A**), relative amplitude (Span index), and the percentage of droplets smaller than 100 µm (**B**). D_v50_ is the droplet diameter such that 50% of the sprayed volume consists of droplets smaller than that value (VMD equivalent).

Variations in the size and spectrum of droplets can affect the results of tests involving pesticide application technology^28^. Spectrum analysis of the droplets formed by the flat fan nozzle utilized in all applications indicated that at the range of the working pressure (2.0 to 2.5 bar or 200 to 250 kPa), the VMD varied from 402.2 to 237.1 µm (equivalent to D_v50_), configuring a coarse to medium droplet size (Fig. 4A), according to the conditions of the fields. This range of droplet size variation is most commonly used for soybean and maize crops in the region. Droplet size is one of the most important parameters in pesticide application programs that may affect the deposits and spray coverage on the bottom leaves of the crop; however, the volume of spray deposits on the soybean canopy had minimal difference regardless of the spray nozzle type^29^. The relative amplitude (span index) indicates the homogeneity of the droplet population: if the spectrum of the droplets has the lowest value, the spectrum is more homogeneous. In this experiment, this variable was stable in the working pressure range.

### Site-specific spraying on the crops

**Figure 5:**
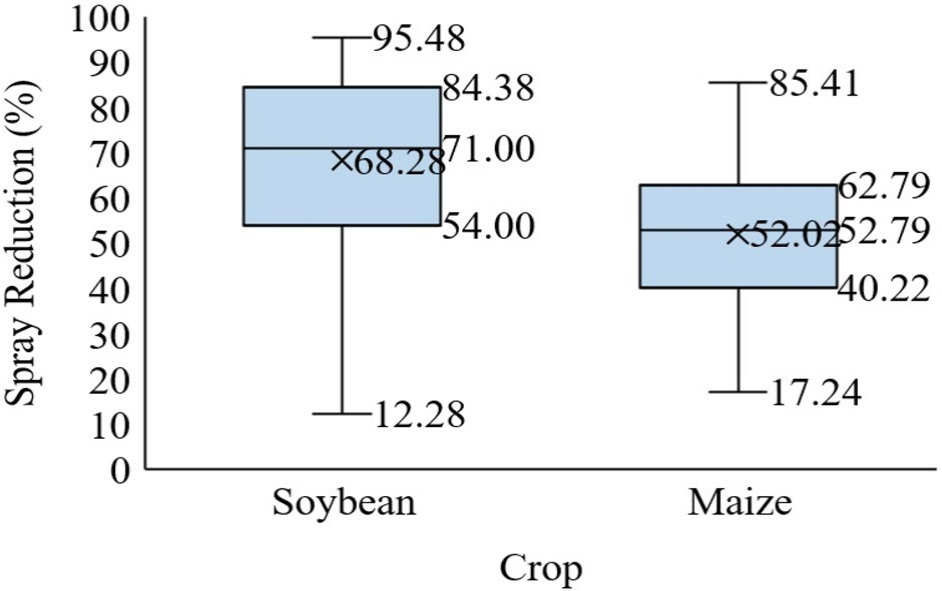
Percentage reduction of pesticides applied using the site-specific spraying equipment with real-time sensors on soybean and maize crops.

The dispersion of the reduction in the volume of pesticide applied in the soybean crop is significantly greater than that in the maize crop (Fig. 5), considering that the type of spray application is different. In addition, the spray applications over the maize were made only in the post-emergence stage of the crop, i.e., when the benefits of the real-time spraying technology seem to be less evident than when the field has no crop. It was observed that in the soybean crop, the variability on reduced pesticide usage ranged from 12 to 96%, and in the maize crop, this variability ranged from 17 to 85%. The greater or lesser reduction in the use of pesticides could also be associated with the architecture of the cropped plant because in greater spacing between rows, there is a more extended time necessary for the complete closure of the crop leaves. The effects of the change in row spacing are associated with yield and changes in crop phenology, such as pod insertion, number of grains per plant, and accumulation of dry matter^30^.

A significant difference in the reduction of pesticides applied by the site-specific equipment was found when comparing the type of spray operation (Table 3) in soybean and maize crops. Thus, spraying operations in both crops allow for a more significant volume reduction in applied pesticides, which is associated with the percentage of vegetation covering the soil, with respect to the total area applied. The smaller the vegetation covering the soil, the more significant is the reduction in the volume of pesticides. Dammer^9^ demonstrated the reduction of herbicides using site-specific spraying technology and reported a reduction of between 30 and 43% in the pesticide volume in carrots. Following the same line of research, Shearer and Jones^31^ obtained 15% savings in the volume of herbicide applied post-emergence on the crops. For using this technology in weed control during the first application on the crop, differences in the reduction of the herbicide volumes depend on several factors, including primarily the weed species present, their growth stage, the climatic conditions at the time of spraying, and the presence of resistant weeds^5^.

**Table 3:** Analysis of variance for the different types of spray operations for soybean and maize crops.

	Degrees of freedom	Medium squares	F value	p > F
**Soybean**				
Treatments	3	7181	33.2	< 0.001
Error	167	216		
**Maize**				
Treatments	3	445	2.8	0.047
Error	85	161		

**Figure 6:**
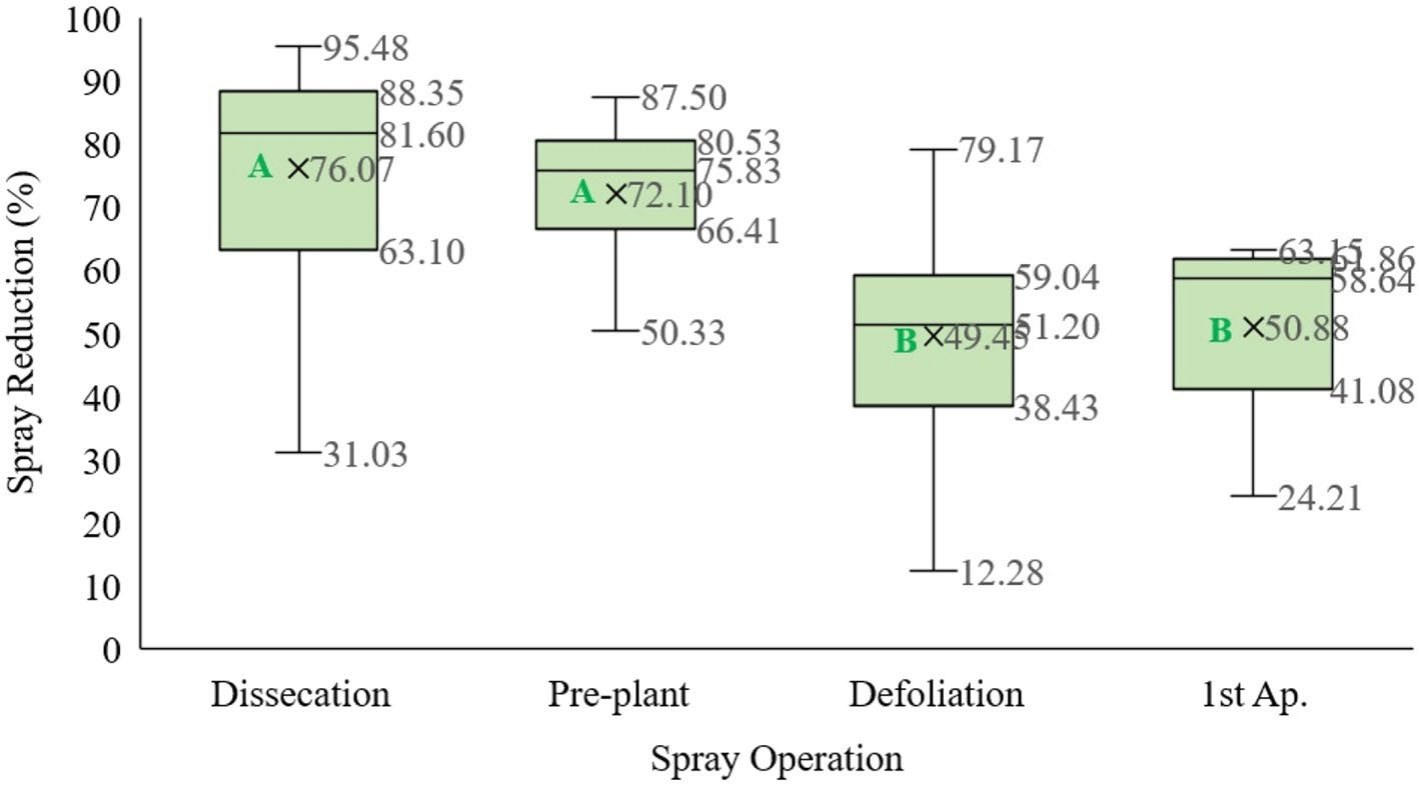
Percentage reduction of pesticide application in different types of operations using the site-specific spraying equipment with real-time sensors on soybean crop. Average values marked with the same letter are significantly similar (Duncan test at 95%).

It was found that desiccation and pre-planting applications showed the highest averages of pesticide reduction, being close to 76.0% and 72.1%, respectively, considering the different spraying operations studied for the soybean crop (Fig. 6). It is important to highlight that these spraying applications occurred before soybean sowing. The sprayed applications after crop sowing showed a lower average reduction in the volume of the pesticides applied compared to desiccation or pre-planting operations, with an average reduction of 51%. Such a decrease in reduction could be attributed to the fact that these applications were made during soybean cultivation, with the row spacing almost closed by plant leaves. With the advance in the development of the crop, an increase in the leaf area and alteration of the plant architecture is normal, contributing to the closing of the row spaces. The soybean crop reaches the maximum growth phase and foliage at 30–35 days after sowing, and reducing row spacing allows an increase in light utilization at the beginning of crop development, leading to a higher leaf area index and biomass production^32^.

Based on the results of pre-harvest defoliation application at the end of the soybean crop cycle, it was possible to detect averages dispersed among themselves from the studied fields, with variations in the volume of reduction ranging from 12 to 79%. This variability may be a consequence of the time of application compared to the crop cycle in each field separately because the site-specific detection sensor does not detect the phenological stage of the plant, rather it checks for the presence of plant leaves. It is also important to note that the soybean crop begins its senescence with spatial variability in the field, which can be due to several field factors, such as severity in the attack of pests and diseases, soil moisture content, and plant nutrition^33^. These characteristics affect the plant foliage, and consequently, the reduction in the volume of pesticides applied by site-specific spraying equipment based on optical sensors.

**Figure 7:**
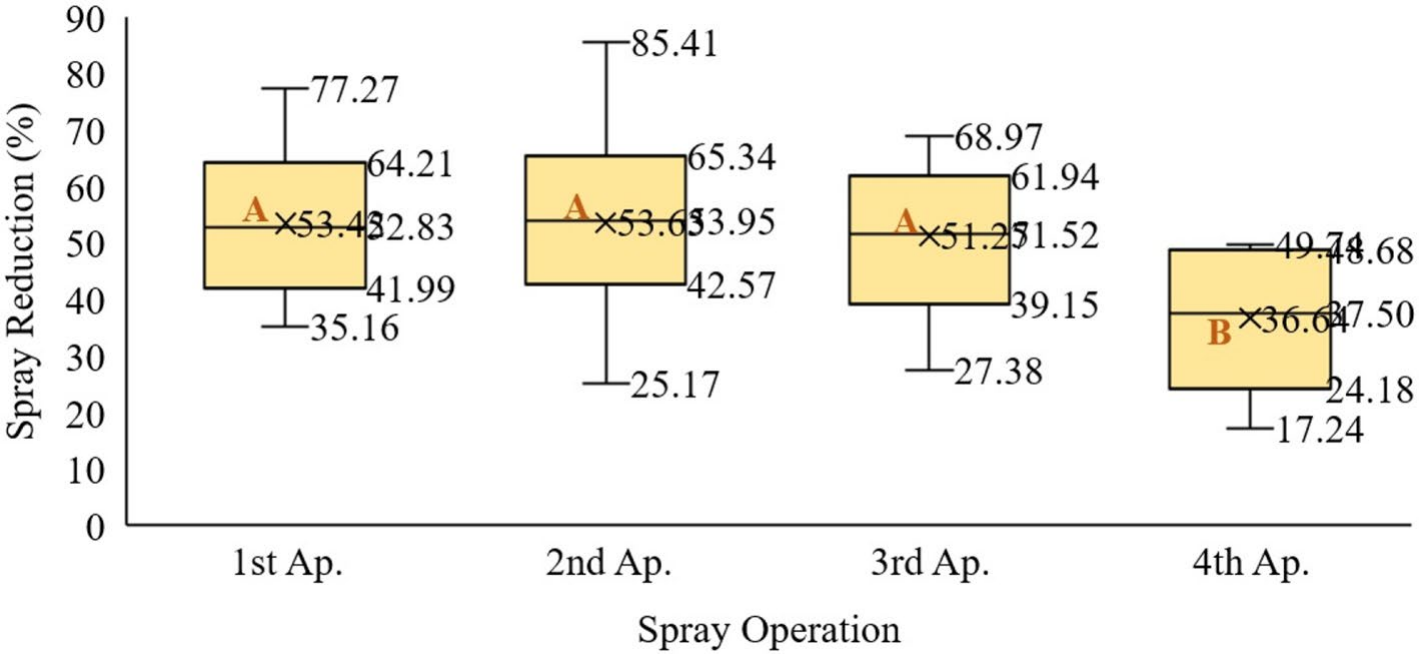
Percentage reduction in pesticide application in different types of operations using site-specific spraying equipment with real-time sensors on maize. Average values marked with the same letter are significantly similar (Duncan test at 95%).

The variability of the volume reduction of pesticides applied to the maize crop ranged from 53.7 to 36.6% (Fig. 7). The results from the first, second, and third spray applications did not differ significantly from each other. The results from the fourth spray application showed an average of 37% (significantly different from the percentages from other spray applications) volume reduction of pesticide application, once the crop developed and increased the foliage, covering the soil on the row spacings. Several studies that have evaluated the utility of this technology to manage weeds have achieved a significant reduction, in herbicide usage levels, of approximately 30–40%^34^. This technology facilitates the use of a practical alternative approach that focuses on precision spraying in the context of environmentally friendly pesticide application. The relationship between row spacing and the phenological growth characteristic of the maize crop can explain the 15% difference in reduction of pesticide application between the third and fourth applications. The lateral spatial resolution of each spray nozzle of the site-specific spraying equipment is 0.20 m; thus, this reduction may have occurred because the length of plant leaves exceeded plant length, forcing the system to activate another nozzle to reach the desired target.

Further investigation is required to determine the extent to which these results can be expanded to other crops, varieties, and types of applications. Other pesticide applications, as well as herbicide applications (for weed patch control), with this site-specific technology, must be studied. Recently, studies on insecticides have gained attention^12^. A study with aphids and ladybird beetles in cereals indicated that site-specific spraying with sensor technology could reduce insecticide use by 13% on average^35^.

### Comparison of crop yield

There was a significant difference between the average yields when comparing the soybean yield on farms using site-specific spraying and using conventional spraying application over the entire field area (Table 4), This difference can be explained by several yield factors, such as climate factors that affect crop growth every year. There was a high yield for crops grown using both styles of pesticide application, corresponding to pesticide treatments in the years 2017 and 2020, and there was a lower yield for crops grown using both styles of pesticide application, corresponding to pesticide treatments in the years 2018 and 2019. As for the maize crop, both spraying methods showed the same average yield across all evaluated years. Based on these results, it can be inferred that the yield of soybean and maize crops was not affected directly by applying the site-specific spraying technology, while reducing the total volume of pesticides sprayed. For Ritter et al.^36^, the low weed densities can explain the large yield of the untreated control at some locations in the experimental field and the absence of negative side effects of the herbicide because it was not sprayed. According to the authors, several factors affect crop yield, which can be used to assess within-field heterogeneity in yield by applying precision farming techniques. Hamouz et al.^37^ also reported that the yield was not lowered by site-specific weed management.

**Table 4:** Average yields of soybeans and maize according to all experimental fields.

Year	System	Crop yield (t ha^−1^)*	
		Soybean	Maize
2017	Conventional	4202.1a	7669.3a
2018	Conventional	3828.8b	7648.6a
2019	Site-specific spraying	3788.6b	7504.6a
2020	Site-specific spraying	4208.1a	6904.1a

*Means with the same letter in the column are statistically similar (95% Duncan test). Twenty-two replicates were considered for each treatment. ANOVA F value: 33.2 for soybean comparison (p-value<0.01); and 2.7 for corn (p-value>0.05).

A reduction in volume of pesticide applied occurred because bare soil was not sprayed and the pesticide was not wasted. This technology does not have the objective, and if it increases the productivity of the crop, however, due to not applying conventionally over the whole field, the question arises if the crop yield could not have been affected.

### Cost of applied pesticides

The cost per area of herbicides was noted to be decreasing over the soybean crop cycle (Table 5), and the cost of insecticides was noted to be increasing. It was also noted that no fungicide spraying was performed on the soybean crop until the crop cycle coincided with the first application, possibly because the crop still did not have enough leafing to create a favorable microclimate for the attack of fungi to their respective phenological stage. As the phenological stages of soybean progress, there is a greater need for insecticides, particularly in the early stages of the reproductive phase^38^. As the crop foliage closes between the lines, the competition between plants and weeds decreases, thereby decreasing the need for herbicides^5^.

**Table 5:** Pesticide costs applied to the soybean crop in two harvest seasons.

Pesticide	Total applied (L or kg)	Total cost (US$)	Total treated area (ha)	Total by area (US$ ha^−1^)*
**Desiccation**				
Herbicide	1730.30	$ 37,128.78	16,595.45	$ 3.73
Insecticide	172.15	$ 549.71	510.00	$ 0.46
**Pre-planting**				
Herbicide	678.83	$ 11,133.49	6098.00	$ 9.18
Insecticide	209.18	$ 11,803.39	2049.00	$ 2.80
**1st Application**				
Herbicide	122.50	$ 850.15	210.00	$ 4.02
Insecticide	111.56	$ 4241.49	1571.00	$ 8.42
**Pre-harvest defoliation**				
Herbicide	1.521.50	$ 4229.77	1399.70	$ 2.52
Insecticide	850.47	$ 11,335.16	4798.70	$ 7.04

*Sum of individual pesticide costs, divided by the specific treated area, as pesticides are more costly than others.

It was possible to verify that there was often a need for more than the same type of spray operation in the same field (Table 5), considering the scenario of the two agricultural years analyzed and considering the local conditions of the farms taken as an experimental field. This result is illustrated by the treated area in some applications (7.404 ha in 2 years) being higher than the sum of the areas on two farms (3.702 ha) when analyzing the volume of pesticides sprayed on the soybean crop in two agricultural years and considering all applications. Some spray applications were not necessary for all fields, totaling a treated area much smaller than the sum of the areas of the two farms. As an example, it can be observed that insecticides, in the first post-emergent application, were applied on only 1571 ha across both farms in two agricultural years. This illustrates that the potential for reducing the volume of pesticides through the use of site-specific spraying equipment can vary, depending on the crop being grown and the condition of the field, possibly in relation to the level of economic threshold of the pest being sprayed.

Herbicide consumption decreased with an increase in the phenological phase of the maize cycle when analyzing the volume of pesticides sprayed on the crop in two agricultural years and considering all applications (Table 6). The total cost of fungicide on maize crops increased significantly from the first to the third application. From 2000 to 2010, foliar fungicides have been increasingly applied to maize, owing to the occurrence of disease pressure; moreover, with shifts to shorter rotations and reduced forms of tillage, residue-borne diseases have started appearing more frequently^39^.

**Table 6:** Pesticide costs applied to the maize crop in two harvest seasons.

Pesticide	Total applied (L or kg)	Total cost (US$)	Total treated area (ha)	Total by area (US$ ha^−1^)*
**1st Application**				
Herbicide	3998.90	$ 22,231.16	4055.40	$ 7.09
Insecticide	5049.41	$ 33,506.56	7766.40	$ 21.37
Fungicide	51.07	$ 1192.71	209.00	$ 3.81
**2nd Application**				
Herbicide	7201.41	$ 21,629.05	6304.60	$ 4.45
Insecticide	1153.53	$ 29,298.00	8062.40	$ 20.21
Fungicide	542.04	$ 8821.49	1680.00	$ 9.80
**3rd Application**				
Herbicide	2745.51	$ 7763.89	2580.60	$ 3.42
Insecticide	971.11	$ 30,413.03	6647.30	$ 30.57
Fungicide	1863.76	$ 19,365.73	4390.20	$ 10.40
**4th Application**				
Herbicide	1053.00	$ 3056.56	703.00	$ 4.94
Insecticide	238.30	$ 7432.10	1334.70	$ 11.99
Fungicide	970.50	$ 6856.64	1660.40	$ 7.58

*Sum of individual pesticide costs, divided by the specific treated area, as pesticides are more costly than others.

### Economical analysis

Partial budget analysis (Table 7) reflects the expected average changes in financial expenses using the new technology during the cropping cycle. The variable costs related to the pesticides were lower with the site-specific application technology, excluding the fixed costs associated with equipment acquisition. Fixed costs by area are also significantly lower than variable costs, illustrating the financial potential for reducing investment in inputs. When all costs were added together, the conventional application by total area spraying was noted to have a cost per area of $ 418.71, whereas the use of the site-specific spray application resulted in a cost per area of $ 183.50, corresponding to a reduction of 56.16%. Published results on the profitability of precision farming techniques can be challenging to interpret due to differences in experimental design and assumptions about included costs^40^.

**Table 7:** Partial budget analysis performed on soybean and corn crops in two harvest seasons, comparing site-specific spraying equipment based on real-time sensors.

Cost factors	Site-specific spraying	Total area spraying
	Cost^3^ (US$ ha^−1^)	Cost^3^ (US$ ha^−1^)
**Fixed cost (equipment)**		
Interest costs^1^	$ 0.45	$ 0.13
Depreciation cost^2^	$ 0.79	$ 0.23
**Variable cost (pesticides)**		
Soybean		
Dissecation	$ 14.12	$ 46.13
Pre-planting	$ 20.66	$ 48.51
1st Aplication	$ 12.44	$ 28.16
Defoliation	$ 9.56	$ 17.25
Maize		
1st Aplication	$ 55.63	$ 69.17
2nd Aplication	$ 54.52	$ 75.28
3rd Aplication	$ 68.4	$ 87.46
4th Aplication	$ 24.51	$ 38.55
Variable cost (pesticides)	$ 182.68	$ 418.71
Additional fixed cost	$ 0.88	–
Total cost	$ 183.56	$ 418.71
Cost reduction (%)	56.16%	

^1^Interest in equipment financing was considered at 6.5% per year in the Brazilian market.

^2^Depreciation of the equipment being considered a useful life of 15 thousand hours.

^3^Sum of individual pesticide costs, divided by the specific treated area, as pesticides are more costly than others.

The potential profitability of selected precision agriculture technologies will undoubtedly depend on many variables such as farm and field size, crop, soil type, degree of specialization at the farm, on-farm labor costs, and access to finance^40^. In addition, individual farmers may decide on adoption depending on their current investments in machinery and the expected time of replacement. Other elements that may hinder or promote adoption are access to training and extension services related to precision farming technologies^35^. The implementation of precision farming promotes the rational application of pesticides; however, it requires capital investment^34^. Farmers should keep in mind a long-term perspective when adopting this technology.

## Conclusions

Site-specific spraying equipment based on real-time sensors provides a reduction in the volume of pesticides used in soybean and maize crops, with respect to the application of herbicides, as well as other agricultural pesticides such as insecticides and fungicides. There was a significant reduction in the volume of the pesticides applied in the post-emergence condition of soybean and maize crops in the initial phenological stages when the crop still had less leaf area or less foliage coverage between the lines. The crop cost reduction using this technology was 56.16%, compared to the cost associated with pesticide application in the total area.

Under the experimental conditions applied, there were no differences in the yields of soybean and maize crops obtained using this technology, compared to the historical yield of soybean and maize crops.

## Supplementary Information

Supplementary Tables.
