# Supplementary material for: Reduction of pesticide application via real-time precision spraying
Source: Sci Rep. 2022 Apr 4;12:5638. doi: 10.1038/s41598-022-09607-w (PMC8980047; doi:10.1038/s41598-022-09607-w)

**Table S1.** Maize addiciontal pesticide cost information spreadsheet.

| Item | Unit | Pesticide | Unit Price Quantity (USD) | | Total Appied | Treated Area  (ha) | Total by Area  (US ha^-1^) |
| --- | --- | --- | --- | --- | --- | --- | --- |
|  | 1^st^ Application | | | | | | |
| Atrazina | L | insecticide | 4,436.11 | $ 2.60 | $11,533.89 | 2,671.40 | $ 4.32 |
| Benzoato de Emamectina | kg | insecticide | 26.50 | $ 52.07 | $ 1,379.86 | 175.00 | $ 7.88 |
| Bifentrina and Carbosulfano | L | insecticide | 11.50 | $14.58 | $ 167.67 | 42.00 | $ 3.99 |
| Carbendazim and Tebuconazol | L | fungicide | 16.00 | $ 6.07 | $ 97.12 | 42.00 | $ 2.31 |
| Clorantraniliprole | L | insecticide | 49.30 | $ 112.82 | $ 5,562.03 | 1,001.50 | $ 5.55 |
| Clorfluazurom | L | insecticide | 21.50 | $14.75 | $317.13 | 113.00 | $ 2.81 |
| Diflubenzurom | L | insecticide | 41.50 | $ 2.83 | $117.45 | 572.50 | $ 0.21 |
| Estrobilurina. Picoxistrobina | L | fungicide | 35.07 | $31.24 | $ 1,095.59 | 167.00 | $ 6.56 |
| Flubendiamida | L | insecticide | 44.55 | $ 100.67 | $ 4,484.85 | 564.00 | $ 7.95 |
| Glifosato | L | herbicide | 3,635.97 | $ 3.12 | $ 11,344.23 | 2,745.90 | $ 4.13 |
| Imidacloprido | kg | insecticide | 222.80 | $ 26.03 | $ 5,799.48 | 1,005.50 | $ 5.77 |
| Imidacloprido and Bifentrina | L | insecticide | 96.36 | $ 22.56 | $ 2,173.88 | 635.50 | $ 3.42 |
| Lambda-Cialotrina | L | insecticide | 61.39 | $ 19.96 | $ 1,225.34 | 765.50 | $ 1.62 |
| Metoxifenozida | L | insecticide | 32.00 | $ 19.44 | $ 622.08 | 175.00 | $ 3.55 |
| Paraquate | L | herbicide | 250.00 | $ 2.78 | $ 695.00 | 262.00 | $ 2.65 |
| Tembotriona | L | herbicide | 112.93 | $ 90.25 | $ 10,191.93 | 1,047.50 | $ 9.73 |
| Zeta-Cipermetrina | L | insecticide | 5.90 | $ 20.83 | $ 122.90 | 45.50 | $ 2.70 |
|  | 2^nd^ Application | | | | | | |
| Aminoácido Acido L-Glutâmico | L | amino acid | 30.00 | $ 17.36 | $ 520.80 | 109.00 | $ 4.78 |
| Atrazina | L | herbicide | 3,576.24 | $ 2.60 | $ 9,298.22 | 3,001.30 | $ 3.10 |
| Azoxistrobina | L | fungicide | 100.65 | $ 3.12 | $ 314.03 | 222.00 | $ 1.41 |
| Azoxistrobina and Ciproconazol | L | fungicide | 30.50 | $31.24 | $ 952.82 | 197.00 | $ 4.84 |
| Benzoato de Emamectina | kg | insecticide | 51.39 | $52.07 | $ 2,675.88 | 438.50 | $ 6.10 |
| Bifentrina and Carbosulfano | L | insecticide | 134.25 | $14.58 | $ 1,957.37 | 422.50 | $ 4.63 |
| Carbendazim and Tebuconazol | L | fungicide | 145.90 | $ 6.07 | $ 885.61 | 334.50 | $ 2.65 |
| Clorantraniliprole | L | insecticide | 46.10 | $ 112.82 | $ 5,201.00 | 1,046.50 | $ 4.97 |
| Clorfluazurom | L | insecticide | 286.58 | $14.75 | $ 4,227.06 | 1,539.00 | $ 2.75 |
| Espinetoram | L | insecticide | 4.30 | $ 130.17 | $ 559.73 | 67.50 | $ 8.33 |
| Estrobilurina. Picoxistrobina | L | fungicide | 167.40 | $31.24 | $ 5,229.58 | 704.50 | $ 7.42 |
| Flubendiamida | L | insecticide | 8.75 | $ 100.67 | $ 880.86 | 210.50 | $ 4.18 |
| Glifosato | L | herbicide | 3,613.46 | $ 3.12 | $ 11,274.00 | 3,001.30 | $ 3.76 |
| Imidacloprido | kg | insecticide | 20.96 | $26.03 | $ 545.59 | 374.00 | $ 1.46 |
| Imidacloprido and Bifentrina | L | insecticide | 442.05 | $22.56 | $ 9,972.65 | 2,536.60 | $ 3.93 |
| Lambda-Cialotrina | L | insecticide | 10.85 | $19.96 | $ 216.57 | 112.20 | $ 1.93 |
| Metoxifenozida | L | insecticide | 20.00 | $19.44 | $ 388.80 | 104.00 | $ 3.74 |
| Novalurom | L | insecticide | 63.00 | $20.83 | $ 1,312.29 | 302.00 | $ 2.54 |
| Tebuconazol and Metominostrobina | L | fungicide | 97.59 | $14.75 | $ 1,439.45 | 222.00 | $ 6.48 |
| Tembotriona | L | herbicide | 11.71 | $90.25 | $ 1,056.83 | 302.00 | $ 3.50 |
| Triflumurom | L | insecticide | 57.80 | $20.83 | $ 1,203.97 | 652.10 | $ 1.85 |
| Zeta-Cipermetrina | L | insecticide | 7.50 | $20.83 | $ 156.23 | 257.00 | $ 0.61 |
|  | 3^rd^ Application | | | | | |  |
| Atrazina | L | herbicide | 1,542.51 | $ 2.60 | $ 4,010.53 | 1,195.30 | $ 3.36 |
| Azoxistrobina | L | fungicide | 881.33 | $ 3.12 | $ 2,749.75 | 1,752.80 | $ 1.57 |
| Benzoato de Emamectina | kg | insecticide | 30.50 | $52.07 | $ 1,588.14 | 190.00 | $ 8.36 |
| Bifentrina and Carbosulfano | L | insecticide | 74.00 | $14.58 | $ 1,078.92 | 210.50 | $ 5.13 |
| Carbendazim and Tebuconazol | L | fungicide | 468.19 | $ 6.07 | $ 2,841.91 | 762.70 | $ 3.11 |
| Clorantraniliprole | L | insecticide | 49.80 | $ 112.82 | $ 5,618.44 | 1,071.50 | $ 5.24 |
| Clorfluazurom | L | insecticide | 234.86 | $14.75 | $ 3,464.19 | 848.80 | $ 4.08 |
| Diflubenzurom | L | insecticide | 26.20 | $20.83 | $ 545.75 | 262.00 | $ 2.08 |
| Espinetoram | L | insecticide | 36.80 | $ 130.17 | $ 4,790.26 | 578.80 | $ 8.28 |
| Estrobilurina. Picoxistrobina | L | fungicide | 375.32 | $31.24 | $ 11,725.00 | 1,543.70 | $ 7.60 |
| Glifosato | L | herbicide | 1,203.00 | $ 3.12 | $ 3,753.36 | 1,385.30 | $ 2.71 |
| Imidacloprido | kg | insecticide | 105.41 | $26.03 | $ 2,743.82 | 1,421.00 | $ 1.93 |
| Imidacloprido and Bifentrina | L | insecticide | 308.21 | $22.56 | $ 6,953.22 | 1,383.30 | $ 5.03 |
| Indoxacarbe | L | insecticide | 61.23 | $45.13 | $ 2,763.31 | 245.70 | $ 10.05 |
| Lambda-Cialotrina | L | insecticide | 18.60 | $19.96 | $ 371.26 | 245.70 | $ 1.51 |
| Metoxifenozida | L | insecticide | 25.50 | $19.44 | $ 495.72 | 190.00 | $ 2.61 |
| Tebuconazol and Metominostrobina | L | fungicide | 138.92 | $14.75 | $ 2,049.07 | 331.00 | $ 6.19 |
|  | 4^th^ Application | | | | | |  |
| Atrazina | L | herbicide | 440.00 | $ 2.60 | $ 1,144.00 | 210.00 | $ 5.45 |
| Azoxistrobina | L | fungicide | 496.00 | $ 3.12 | $ 1,547.52 | 745.70 | $ 2.08 |
| Carbendazim and Tebuconazol | L | fungicide | 378.00 | $ 6.07 | $ 2,294.46 | 535.70 | $ 4.28 |
| Clorfluazurom | L | insecticide | 96.00 | $14.75 | $ 1,416.00 | 290.00 | $ 4.88 |
| Espinetoram | L | insecticide | 25.30 | $ 130.17 | $ 3,293.30 | 299.00 | $ 11.01 |
| Estrobilurina. Picoxistrobina | L | fungicide | 96.50 | $31.24 | $ 3,014.66 | 379.00 | $ 7.95 |
| Glifosato | L | herbicide | 613.00 | $ 3.12 | $ 1,912.56 | 493.00 | $ 3.88 |
| Imidacloprido | kg | insecticide | 24.00 | $26.03 | $ 624.72 | 290.00 | $ 2.15 |
| Imidacloprido and Bifentrina | L | insecticide | 93.00 | $22.56 | $ 2,098.08 | 455.70 | $ 4.60 |

**Table S2.** Soybean addiciontal pesticide cost information spreadsheet.


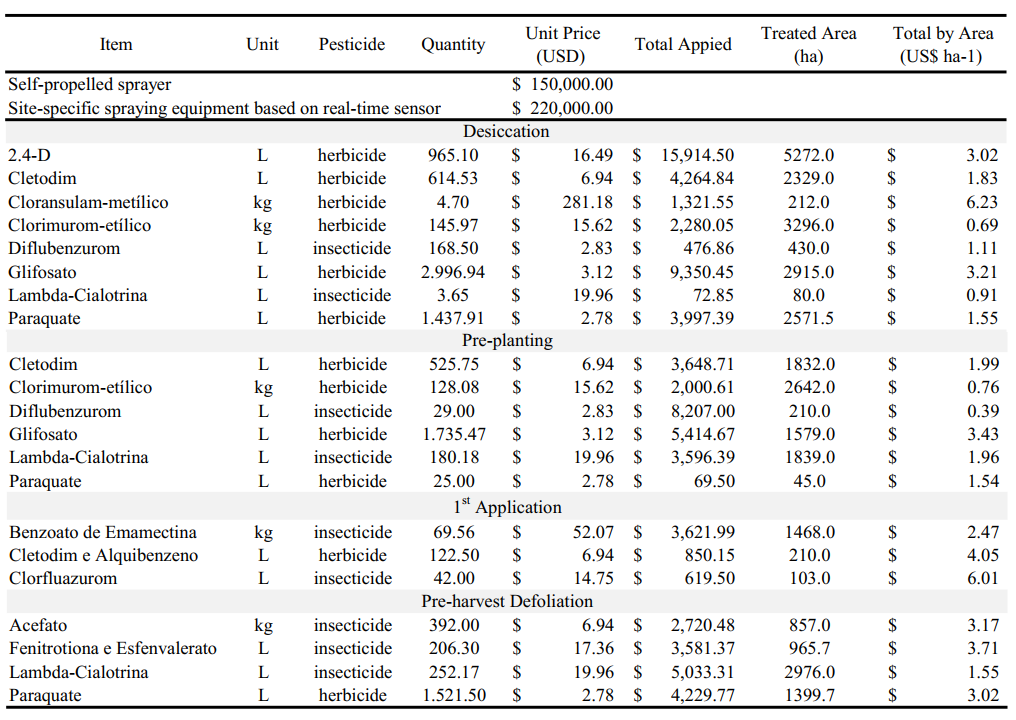

Supplement: Supplementary file 1 — Supplementary Tables. [file 41598_2022_9607_MOESM1_ESM.docx]
